# A Rare Case of Recurrent Carcinoma Ex Basal Cell Adenoma of the Parotid Gland

**DOI:** 10.7759/cureus.46891

**Published:** 2023-10-12

**Authors:** Rajab A Alzahrani

**Affiliations:** 1 Division of Otolaryngology, Department of Surgery, College of Medicine, Al-Baha University, Al-Baha, SAU

**Keywords:** salivary gland carcinoma, surgical excision, head and neck tumor, basal cell neoplasm, salivary gland

## Abstract

Basal cell adenoma is encountered in the salivary glands, particularly the parotid gland; however, malignant transformation is rare, and recurrence is much rarer. We report the case of a 60-year-old man who had experienced a slow-growing mass in the parotid gland, which was suspected to be pleomorphic adenoma. Radiological and cytological examination suggested an atypical lesion in the left parotid. The final diagnosis reached on the excised specimen was that of a basal cell adenocarcinoma ex adenoma with close margins. The patient presented with a recurrence after two years. Routine histopathological examination with careful examination by the pathologist is essential for proper management of such rare malignant lesions, and recurrence is a possibility. A complete excision of the tumor with tumor-free margins from the beginning is suggested.

## Introduction

Basal cell adenoma (BCA) is a rare benign neoplastic lesion of the salivary gland, accounting for 1-7.5% of all salivary gland tumors, and is more frequently seen in elderly patients, with a female predilection [1]. BCA usually arises in the parotid gland, with very few reported cases in minor salivary glands, mainly at the submandibular gland [2].

It is challenging to differentiate the benign lesion, BCA, from the other potential differential diagnoses including benign and malignant lesions, such as biphasic salivary tumors (pleomorphic adenoma, adenoid cystic carcinoma, epithelial myoepithelial carcinoma, and basal cell adenocarcinoma), and the malignant transformed tumor "carcinoma ex pleomorphic adenoma” [3]. Very few cases of basaloid tumors in the salivary glands have been reported, with almost all cases being benign (adenoma) [4]. The malignant transformation of benign tumors of the salivary gland is predominantly seen in the pleomorphic adenoma, leading to the carcinoma ex pleomorphic adenoma. This malignant transformation from the pleomorphic adenomas has been observed to range from 4.5% to 8.5% of cases [5]. However, carcinomas arising in BCA are rare with few reported cases arising in BCAs with different malignant components including adenocarcinoma not otherwise specified, salivary duct carcinoma, and basaloid carcinoma [6,7].

The treatment of choice for basal cell adenocarcinoma is wide local excision. Since these cancers seldom metastasize to lymph nodes, neck dissection is not routinely recommended. However, reported local recurrence rates of basal cell carcinoma are high, and the mortality rate is low [8,9]. Here we report a rare and challenging case of carcinoma ex BCA with local recurrence within two years to raise the awareness of both surgeons and pathologists of such an unusual tumor disorder.

## Case presentation

**Figure 1:**
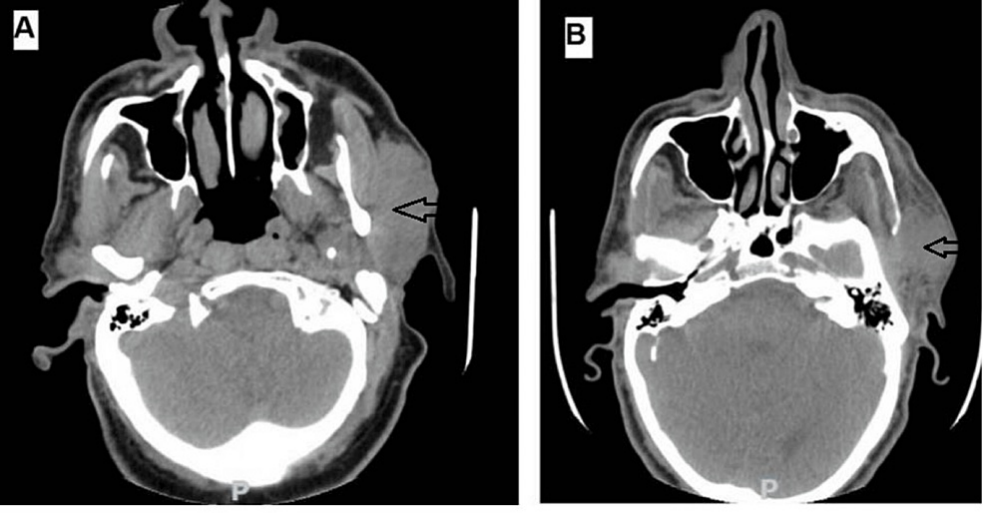
CT showing the masses (arrows) in the parotid gland. (A) Primary lesion. (B) Recurrent lesion. CT, computed tomography

**Figure 2:**
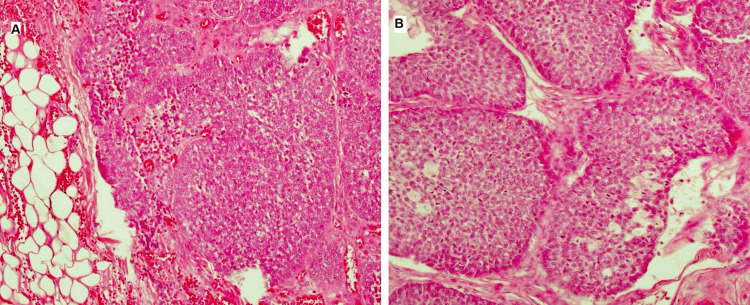
Histopathology picture of the basal cell carcinoma showing atypical basaloid cells (100x H&E). (A) Hemorrhage and invasion. (B) Palisading at the periphery of the histologic nodules.

A 60-year-old man presented with a slow-growing slightly painful mass in his left parotid gland. Physical examination at the otorhinolaryngeal clinic revealed a freely movable mass measuring 55 x 50 mm, without fixation to the adjacent tissue with an intact facial nerve. A clinical suspicion of pleomorphic adenoma was made, and the patient was advised to undergo ultrasound and CT examinations, which revealed a well-demarcated mass (Figure 1). Fine needle aspiration (FNA) cytology was performed, which showed hypocellular smears of irregular cohesive cell clusters with non-fibrillary stroma and very occasional basaloid-like cells with atypia (Milan System for Salivary Gland Cytopathology Reporting, category III). Parotidectomy was performed after intradepartmental consultation. During the operation, a purplish to pinkish encapsulated and fairly defined tumor was found located in the deep side of the deep lobe. The specimen was sent for histopathological examination, which rendered a surgical pathology report indicating basal cell carcinoma ex BCA depending on the microscopic examination showing multifocal tubular, trabecular, and solid growth patterns with peripheral palisading abutting the surrounding normal tissue (Figure 2). The tumor showed occasional hemorrhage and focal tumor necrosis with cellular features of basal cell tumor including hyperchromatic, round to oval, mitotically active, pleomorphic, and vesicular nuclei, in addition to diminished cytoplasm. The tumor was also surrounded by thin fibrous tissue and basaloid cells with regular outlines arranged in a mainly a trabecular pattern, suggesting a transition from this lesion to malignancy. No vascular permeation was seen. Immunohistochemical staining revealed positive pancytokeratin, p63, and calponin; however, beta-catenin was negative according to the pathology report. Close surgical margins (less than 1 mm) were also seen. The patient was advised for completion operation but was unfit according to the anesthetists and was thus referred to the oncology department. Two years following the primary operation, the patient presented with a mass lesion. CT examination revealed a fairly defined mass abutting the masseter muscle and extending to the infratemporal fossa, with some infiltration to the fat. The new mass at the site of the previous operation was biopsied, which revealed a recurrence of the primary tumor.

## Discussion

Basal cell adenocarcinoma and BSA represent basaloid neoplasms that occur primarily in the parotid gland in older individuals worldwide; however, their occurrence in the minor mucosal salivary gland has been rarely described [10]. Kleinsasser et al. in 1967 described the neoplastic lesion, BCA. Following this nomenclature and description, these unusual lesions were increasingly being reported as tumors, with specific features distinguishing them from the commonly presented lesion, pleomorphic adenomas [11].

The basal cell lesions usually arise from the intercalated ductal structures; distinct morphologic features matching those cells can be divided into intercalated duct hyperplasia and intercalated duct adenoma resembling the BCAs [12].

BCA is a rare but benign type of salivary gland neoplastic lesion that displays relatively high nucleus ratios, is cribriform and solid, and proliferates to produce trabecular and membranous patterns, but cellular atypia can also be seen in occasional cases. Around half of BCAs carry b-catenin gene (*CTNNB1*) mutations. Therefore, the tumorigenic process for the tumor may be related to the known signaling pathway *CTNNB1-Wnt* in addition to its relationship with the *CYLD* tumor suppressor gene [4].

The malignant transformation is a rare event in cases of BCA and most commonly reported in cases of adenoid cystic carcinoma, carcinoma ex monomorphic adenoma, and basal cell adenocarcinoma [4,13].

It is not easy to detect the malignant transformation of BCA as the clinical, radiological, and histological features differentiating adenomas (including BCA) from basal cell carcinoma are difficult to separate by ordinary studies alone, even after excision and microscopic examination, as both benign and malignant lesions are composed of basaloid cells with different grades of atypia [10,14]. In this case, we tried FNA cytological examination, which showed inconclusive cytological features; however, atypia appeared. From a cytological point of view, the cells of both benign and malignant lesions are essentially indistinguishable, which makes FNA separation of the two tumors impossible in most cases [15].

The invasion of surrounding glandular tissue and other soft tissues is the most critical feature to distinguish basal cell adenocarcinoma from benign lesions; however, the morphologic separation, depending on the histopathology of BCA and basal cell adenocarcinoma, can also be difficult, and immunohistochemistry is required [16]. In this case, invasion was detected by the local pathologist taking a remote consultation with a head and neck histopathologist aided by a few immunohistochemical markers.

Histopathology examination is the gold standard diagnostic tool for most challenging head and neck carcinomas, and immunohistochemistry and ancillary tests are commonly used for accurate diagnosis in addition to recently used telepathology and remote consultation technology for the different types of carcinomas [10,17,18].

The best surgical approach for such rare tumors is still unclear. Some authors recommended parotidectomy or local excision depending on tumor size (more or less than 4 cm) and extraglandular tumor extension.

Surgery with clear, free surgical margins is always the goal of all head and neck surgeons and is the curative method in the majority of cases [19]. In our case, the primary surgery was performed with close margins due to a lack of definitive diagnosis and staging, and the patient came back with a local recurrence within two years.

Lymphadenectomy may be a surgical option for patients showing clinically involved nodes and/or radiological suspicion for node extension. Nodal dissection is well established in other types of malignant lesions; therefore, it should also be considered in this rare type of lesion (basal cell carcinoma). This surgical procedure may have some consequences on the long run, resulting from the facial nerve anatomical course that might be involved. Therefore, nerve injury complications, wound dehiscence, epidermolysis, or leak of chyle can occur [20].

The role of radiotherapy also remains controversial in the case of basal cell adenocarcinomas; however, some authors recommend radiation after surgery in patients with high-risk features only, such as perineural and lymphovascular invasion, local recurrences, minor gland affection, and close surgical margins [8,20].

## Conclusions

Clinical manifestations and radiological features have several limitations in differentiating salivary gland basal cell tumors. The rare lesion basal cell adenocarcinoma is a histological diagnosis and should be suspected even in slowly progressive parotid lesions. The surgical excision with clear margins is essential to prevent local recurrence, which is not unusual.
